# A New Marker of Myocardial Bridge?

**DOI:** 10.5935/abc.20180264

**Published:** 2019-01

**Authors:** Paolo Blanco Villela, Gláucia Maria Moraes de Oliveira

**Affiliations:** 1 Universidade Federal do Rio de Janeiro (UFRJ), Rio de Janeiro, RJ - Brazil; 2 Instituto do Coração Edson Saad - UFRJ, Rio de Janeiro, RJ - Brazil

**Keywords:** Myocardial Bridging/physiopathology, Inflammation, Atherosclerosis, Risk Factors, Monocytes, Lipoproteins, HDL

Myocardial bridges (MBs) have been associated with an increased incidence of cardiovascular events. Even though the pathophysiology of this association is still elusive, it seems to be related to the development of atherosclerosis. This hypothesis is based on changes in blood flow resulting from systolic compression of coronary arteries and leading to changes in arterial wall shear stress, which could act as a proatherogenic event affecting the endothelium of the arterial segment proximal to the MB.^1^

Recently, Akishima-Fukasawa et al.^2^ assessed 150 autopsies of individuals without cardiovascular heart disease to verify the influence of MBs on the development of atherosclerosis. The authors found the occurrence of MBs in 93 hearts, and using computer-assisted histomorphometry, observed a higher frequency of luminal stenosis in segments proximal to the MB. Using a multiple comparison test, the authors showed a relation between the presence of risk factors like hypertension, diabetes, and dyslipidemia to a higher rate of stenosis affecting segments located 2.5 cm proximally to the MB. Despite the absence of MB flow evaluation, the authors documented an anatomical association between significant atherosclerotic lesions and the presence of MBs.^2^ We could speculate that this may be one of the explanations for the increased incidence of cardiovascular events in patients with MB.

The detection of MBs by imaging methods has increased with the use of cardiac computed tomography, which allows for multiplanar and three-dimensional evaluations. Studies using this method have reported prevalence rates of MBs ranging from 5% to 76%, depending on the population studied and the type of MB and equipment used for its detection.^3^^,^^4^ However, the availability of this method for clinical use is limited.

In this issue of the *Arquivos Brasileiros de Cardiologia*, Enhos et al.^5^ proposed that a newly described tool for the assessment of inflammation and atherosclerosis - the relationship between monocytes and high-density lipoprotein (HDL), abbreviated as MHR (monocyte-to-HDL-cholesterol ratio) - could be associated with atherosclerosis in segments proximal to an MB. The authors studied 160 patients with MBs and without significant coronary lesions and observed that at a cut-off value of 13.55, the MHR was able to detect the presence of MBs with a 59% sensitivity, 65% specificity, and an area under the ROC curve of 0.687 (95% confidence interval 0.606-0.769, p < 0.001). On multivariate analysis, the MHR emerged as an independent risk factor for the presence of MB. The authors attributed this association to the occurrence of endothelial dysfunction and inflammation in patients with MB.^5^

Monocytes have a fundamental role in the inflammatory cascade and participate actively in the development and progression of the atherosclerotic plaque, while HDL particles behave otherwise, reducing the expression of tissue factor in monocytes, hindering cell migration and LDL oxidation in the vascular wall.^6^ Observations from biochemical assays have allowed a better understanding of the complex interactions between monocytes and HDL on atherogenesis, and both of them combined seem more appropriate to assess inflammation when compared with the measurement of each of them alone.^6^

The MHR has been associated with different clinical conditions with pathophysiological bases that include an inflammatory component. In regard to chronic coronary disease, Korkmaz et al.^7^ studied 301 patients with intermediate lesions undergoing functional evaluation by fractional flow reserve (FFR) and found that those with an FFR ≤ 0.8 showed the highest MHR values (11.6 ± 3.3 vs. 12.6 ± 2.5, respectively)^7^. In another study, Akboga et al.^8^ assessed the relationship between MHR and the SYNTAX score. In the study, which included 1229 patients, the highest MHR values were found in patients with a score equal to or greater than 23, demonstrating an association with the burden of atherosclerotic disease, according to the interpretation of the authors.^8^ The study by Enhos et al.^5^ excluded patients with known atherosclerotic disease, and since the authors found a positive association between MHR and MB, the inflammation present in both conditions seems to be the likely link.

In addition to being a marker associated with the presence of MB, the MHR has also demonstrated a possible association with prognosis in patients with this condition. In a study with 1598 patients with ST-elevation acute myocardial infarction, the group with the highest MHR tertile (30.1 ± 10.5) showed higher mortality and higher incidence of cardiovascular events during hospitalization and along a 5-year follow-up.^9^ The authors concluded that the MHR was an independent predictor of prognosis in these patients.^9^ Similar findings have been reported by Cetin et al.^10^ in a study with 2661 patients. The authors found an association between higher MHR and increased mortality, as well as an increased rate of stent thrombosis. The study by Enhos et al.^5^ does not allow conclusions about the prognosis of the patients.

In addition to coronary disease, studies have associated higher MHR values to diabetic nephropathy (in patients with diabetic nephropathy compared with healthy subjects and with patients with diabetes and without proteinuria),^11^ presence and severity of metabolic syndrome,^12^ presence of coronary ectasia,^13^ cardiac syndrome X,^14^ and smoking.^15^ Even though these results derived from observational studies, they suggest a relationship between conditions that cause vascular inflammation and MHR changes.

The study by Enhos et al.^5^ has several limitations, including a small cohort derived from a single center and an assessment of the MHR measured transversely, although no relevant differences were observed between individuals with MB and controls regarding factors that are known to influence the MHR. Additionally, other potential causes of alterations in this relationship were not evaluated, such as the practice of physical activity, diet, smoking, and the presence of other underlying inflammatory processes.

However, a great merit of the study is to present further evidence of a possible association between MBs and inflammation, which should be further evaluated in longitudinal studies verifying if the association relates to increased cardiovascular outcomes. Until such data become available, modification of diagnostic and therapeutic approaches based on the routine determination of this association in patients with MB does not seem relevant, considering that most MBs have a benign course and their treatment is still controversial.
